# Previous bottlenecks and future solutions to dissecting the *Zymoseptoria tritici*–wheat host-pathogen interaction

**DOI:** 10.1016/j.fgb.2015.04.005

**Published:** 2015-06

**Authors:** Jason J. Rudd

**Affiliations:** Department of Plant Biology and Crop Science, Rothamsted Research, Harpenden, Herts AL5 2JQ, UK

**Keywords:** Plant pathogen, Dothideomycete, Plant defence, Virulence

## Abstract

*Zymoseptoria tritici* (previously *Mycosphaerella graminicola*, teleomorph, *Septoria tritici*, anamorph) causes Septoria tritici blotch, one of the most economically important diseases of wheat (*Triticum aestivum*). The host pathogenic interaction, as currently understood, is intriguing, and may distinguish *Z. tritici* from many of the current models for plant pathogenic fungi. Many important questions remain which require a deeper understanding including; the nature and biological significance of the characteristic long latent periods of symptomless plant infection; how/why the fungus then effectively transitions from this to cause disease and reproduce? Elements of this transition currently resemble a putative “hijack” on plant defence but how is *Z. tritici* able to do this without any form of plant cell penetration? This commentary provides a summary of the recent history of research into the host-pathogen interaction, whilst highlighting some of the challenges going forwards, which will be faced by improved technologies and a growing research community.

## Highlights

• Fungal infection of plants with long latent periods.
• Fungal manipulation of plant defences.
• Models for strictly extracellular mode of pathogenesis.
• Current status of the Zymoseptoria vs wheat interaction.

This commentary on the *Zymoseptoria tritici* vs. wheat host-pathogen interaction seeks to address progress that has been made in the last decade of research, dating from approximately 2004. The main molecular genetic resources available at that time to study the susceptible interaction included two variable size fungal EST collections (Keon et al., 2005a; Kema et al., 2008), an approximate predicted one quarter genome cDNA microarray (Keon et al., 2005b), and ∼300,000 wheat ESTs (mostly not from leaves), some of which were just being used to fabricate early wheat microarrays (http://www.plexdb.org/). However, some very useful experimental tools/procedures had already been developed, in particular the Agrobacterium-mediated fungal transformation procedure (Zwiers and De Waard, 2001), which facilitated the first direct identification of a virulence gene from *Z. tritici*, namely the ABC transporter, MgAtr4 (Stergiopoulos et al., 2003). For wheat, reverse genetics was not so “easy”, and whilst stable transformation with RNAi constructs was being developed it was, and remains, a relatively time consuming methodology, which had not really addressed many pathogenic interactions.

**Fig. 1:**
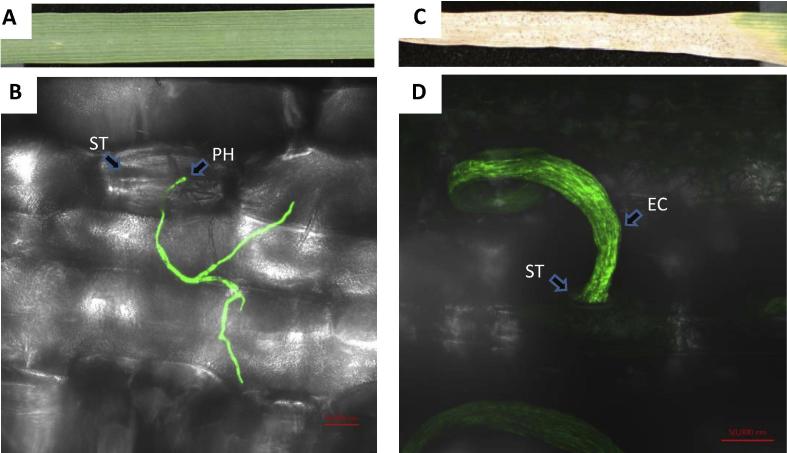
*Z. tritici* uses a strictly extracellular mode of plant pathogenesis with a long latent period for disease development. (A) Susceptible wheat leaf infected with a GFP expressing isolate of *Z. tritici* at 1 day post surface inoculation (1-dpi). (B) Stereomicroscope image of GFP tagged hyphal filaments developing on the leaf surface and penetrating the leaf through a stomatal aperture. Image taken at 1-dpi. ST = stomatal aperture; PH = penetrating hyphae. (C) Susceptible wheat leaf infected with a GFP expressing isolate of *Z. tritici* at 21 day post inoculation (21-dpi). (D) Stereomicroscope image of GFP tagged asexual spore masses exuding within a cirrus from below a leaf stomata. Image taken at 21-dpi. ST = stomatal aperture; EC = extracellular oozing cirrus containing new asexual pycnidiospores.

This article covers only the susceptible disease interaction between *Z. tritici* and wheat and will not address resistant cultivar interactions, which are described elsewhere in this issue. For the “compatible” (or susceptible) interaction alone poses many intriguing questions still pertinent today. Many of these concern the extensive “latent period” of symptomless fungal colonisation, described as the period of time from which the fungus arrives on the host plant (inoculation) to the time when disease symptoms are macroscopically visible and sporulation has commenced (Leonard and Mundt, 1984). Long latent periods are now recognised as quite a conserved and peculiar feature of plant infection by most *Mycosphaerella* fungi, and can in some cases extend to several months. What purpose this serves, its genetic and biochemical basis, and why and how it suddenly ends with the induction of plant cell death remain some of the most scientifically interesting questions for this pathosystem, and others involving related *Mycosphaerella* fungi. This is even more remarkable when you also consider that *Z. tritici* does not appear to penetrate plant cells at any point during infection (Kema et al., 1996), instead exclusively colonising the intercellular spaces following initial entry through plant stomata right through to its exit, via the same route (Fig. 1). There is no such thing as “typical” for this system, but the symptomless latent period for *Z. tritici* on susceptible wheat is usually in the region of 7–14 days prior to leaf cells dying and the onset of fungal asexual sporulation. This suggests exquisite and dynamic communication mechanisms must exist throughout the interaction, and that the onset of wheat cell death is tightly regulated both temporally and spatially (Dean et al., 2012).

The overriding consensus a decade ago was that localised plant cell death in response to microbial pathogens functioned only as an exquisitely organised plant disease resistance response (Heath, 2000), which it probably is against biotrophs and some hemibiotrophs. In contrast cell death occurring during infection by necrotrophs and/or other hemibiotrophs was just some form of random disorganised collapse under the attack of arsenals of pathogen-derived hydrolytic enzymes and toxins. The aforementioned work from Kema and associates (1996) on the histology of infection had already shown that *Z. tritici* did not extensively penetrate plant cells during infection. But it still elicited plant cell death somehow from its extracellular location, and also took over a week to do it. This excellent study was performed using traditional scanning and transmission electron microscopy and first identified some form of host cell perception of fungal hyphae taking place around the onset of plant cell death. This was associated with specific sub-cellular alterations including the movement of particular organelles towards hyphae and irregular enlargement of chloroplast structures. This suggested that the plant cells had begun to “recognise” and respond to something associated with the encroaching extracellular fungal hyphae. Exactly what factors might be recognised is still unknown although a consensus is emerging in several labs that perhaps host selective protein toxins produced in a temporally regulated manner on the switch to necrotrophy might play a role. This would represent a slightly modified model to what has been shown for the wheat pathogens *Stagonospora nodorum* and *Pyrenophora tritici-repentis* in particular (Oliver and Solomon, 2010; Winterberg et al., 2014). This model is currently being tested for *Z. tritici* (see Ben M’Bareck et al., 2015 and Gohari et al., 2015), but it is clear for the plant side of the interaction that the transition to disease symptoms involves very specific changes in gene expression and the activation of signalling pathways which are more commonly associated with plant “defence” (Keon et al., 2007; Rudd et al., 2008, 2015; Yang et al., 2013). Wheat leaf cells ultimately appear to undergo a form of regulated programmed cell death (PCD) in the vicinity of *Z. tritici* hyphae (Keon et al., 2007), in response to these as yet unidentified pathogen cues. The fact that the pathogen effectively reproduces (asexual sporulation) in this environment suggests that the plants effort to “defend” itself has in some way been manipulated or “hijacked” by the pathogen to support its asexual reproduction. This is an attractive model but one which admittedly requires further testing.

Arguably the most significant recent progress has been made in the area of *Z. tritici* genomics. The first publically available genome resource for *Z. tritici* resulted from the actions of a research community led by Dutch and US scientists who lobbied for some time to get a reference genome of *Z. tritici* sequenced and assembled. The case was supported, eventually, by re-emphasizing the point that *Z. tritici* belonged to one of the largest groups of plant pathogenic fungi, the *Dothideomycetes*, and that numerous *Mycosphaerella* species within this group were responsible for causing many of the world’s most important crop diseases (Goodwin, 2004). Despite this there were few available sequenced genomes covering these organisms at that time (Goodwin, 2004). Moreover the sequencing and assembly, done in collaboration with the United States Department of Energy-Joint Genome Institute (US DOE-JGI) was such a great success that the project was extended to produce a “finished” genome of the reference isolate, IPO323 (Goodwin et al., 2011). The high quality of this reference genome (http://genome.jgi-psf.org/Mycgr3/Mycgr3.home.html) has since facilitated comparative studies with sequences from closely related species (Stukenbrock et al., 2011), along with members of the larger *Dothideomycete* class in general (Ohm et al., 2012) with the variable aims of understanding host adaptation, evolution and virulence mechanisms. Of the many notable observations arising from these genome sequences was that *Z. tritici,* and *Mycosphaerella* species in general, have relatively low numbers of predicted secreted plant cell wall attacking enzymes (Goodwin et al., 2011; do Amaral et al., 2012). This is intriguing and may have evolved as either a cause or a consequence of their extracellular lifestyles on their hosts.

It is arguably the search for new virulence and pathogenicity genes which will potentially gain most from the new tools for *Z. tritici* which are described in the range of accompanying articles. Relatively speaking, and despite its agricultural importance, the number of genes which have been shown to contribute to pathogenicity and virulence of *Z. tritici* is small (Table 1 lists 17 to date). Moreover this list is largely made up of global regulators of metabolism and cell signalling including components of mitogen-activated protein kinase pathways and cyclic nucleotide signalling for example. Only one secreted protein effector currently makes this list, the chitin binding protein 3LysM (Marshall et al., 2011). Many, if not all, of these genes have already been ascribed virulence roles in other plant pathogenic fungi (Perez-Nadales et al., 2014), which highlights that the unique determinants that allow *Z. tritici* to colonise wheat leaves remain to be discovered (if they exist at all). Overall this relatively small number of pathogenicity and virulence genes compares quite unfavourably with the numbers reported in literature for the more established models including, for example, *Magnaporthe oryzae, Fusarium graminearum* and *Ustilago maydis*. When you consider that the diseases caused by these fungi still affect global agriculture, this only serves to emphasize the task in hand for *Z. tritici*.

What has been the reason behind such comparatively slow progress in building a catalogue of virulence / pathogenicity genes from *Z. tritici* and corresponding wheat genes which function during the interaction? I would argue that the relatively small size of the research community has played the greatest role, so it is now exciting to see a significant increase in the number of groups now undertaking research on this system from both fungal and plant perspectives. Whilst well established methods to generate *Z. tritici* mutant strains have been around and used for some time (Zwiers and De Waard, 2001; Motteram et al., 2009), including the availability of a Ku70 modified strain of IPO323 (Bowler et al., 2010), there was room for further improvement, particularly in generating gene disruption/deletion constructs and performing parallel fungal transformations in higher throughput. Similarly, the recent development of a Barley Stripe Mosaic Virus (BSMV) – viral induced gene silencing system (VIGs) system for wheat which can be combined with *Z. tritici* inoculations promises to rapidly speed up gene discovery in the host plant, as exemplified by five published examples in the last 2 years alone (Table 1).

However, possibly the single most important technical element which has to date been lacking from studies on the host-pathogen interaction, is the use of advanced cell biological techniques. From the many welcome technical advances described in this issue, I believe that improved cell biology may have the greatest impact. To substantiate this I will offer one example where we understand reasonably well the genetics of the interaction, but where their currently exist gaps in the cell biology. Together with collaborators we had previously demonstrated the importance for *Z. tritici* of evading host recognition through its cell wall chitin, by way of producing a specific effector protein, during the early phases of leaf infection (Marshall et al., 2011). Recent data suggest the genetics of this interaction involve as few as three genes in total which can dictate whether a leaf becomes diseased or resists infection. These include the secreted (or more accurately predicted secreted) chitin binding *Z. tritici* effector 3LysM, which is a homologue of the secreted LysM effectors ECP6 from *Cladosporium fulvum* (Bolton et al., 2008; de Jong et al., 2010; Sánchez-Vallet et al., 2013) and Slp1 from *Magnaporthe oryzae* (Mentlak et al., 2012), along with two predicted wheat plasma membrane receptors TaCEBiP and TaCERK1 (Marshall et al., 2011; Lee et al., 2014). The available genetic and biochemical data suggests that 3LysM sequesters elicitor active chitin fragments in the wheat leaf apoplast and prevents activation of the two putative wheat chitin receptors. This supports the symptomless phase of infection by preventing premature activation of selective plant defences. A similar observation was also made for the interaction of Slp1 from *M. oryzae* with CEBiP from rice (Mentlak et al., 2012) highlighting conservation of an important virulence mechanism.

The fact that as few as three genes can dictate the outcome of the *Z. tritici* on wheat interaction is quite remarkable and clearly manipulation of chitin signalling represents a possible future target for *Z. tritici* control. Nevertheless there are still many gaps in this story which could be addressed with advanced cell biology including, somewhat obviously, demonstrating that all three proteins localise as predicted. But perhaps a more interestingly question would be to determine the precise spatial and temporal dynamics of localisation of the three proteins. For example, exactly where during colonisation of leaf tissue do the *Z. tritici* hyphae need to effectively hide their chitin? Performing such an experiment represents a significant technical challenge which I hope some of the accompanying articles might address. A key consideration will be to demonstrate how deep within infected wheat leaves we can go and still visualise in real-time the dynamics of host and pathogen protein production during the various phases of the interaction? I hope this proves technically possible as it would then allow us to address some rather fundamental questions relating to the effector biology of *Z. tritici* and putative host resistance towards it. For example do exclusively non-cell penetrating pathogenic fungi like *Z. tritici* produce effectors which are internalised into plant cells or not? The published data on disease resistance interactions involving *C. fulvum* (a *Mycosphaerella* fungus with a non-cell penetrating mode of pathogenesis) on tomato may suggest not, as all published resistance genes recognising *C. fulvum* avirulence effectors encode plasma membrane localised (predicted) proteins (Stergiopoulos and de Witt, 2009). This special issue describes many new cell biology and other tools which will soon be available to an expanded research community and will help test this and many other hypotheses, further establishing the *Z. tritici* – wheat interaction as an emerging model system and leading to future applications relevant to disease control.

## Figures and Tables

**Table 1:** Current list of *Z. tritici* and *T. aestivum* genes which influence the outcome of the susceptible host-pathogen interaction.

*Z. tritici* gene name	Gene function	Reference	*T. aestivum* gene name	Gene function	Reference
***Atr4***	ABC Transporter	Stergiopoulos et al. (2003)	***CERK1***	Putative chitin activated receptor kinase- competes genetically with *Z. tritici****3LysM***	Lee et al. (2014)
***Fus3***	Mitogen-activated protein kinase (MAPK)	Cousin et al. (2006)	***CEBiP***	Putative chitin binding protein- competes genetically with *Z. tritici****3LysM***	Lee et al. (2014)
***Slt2***	Mitogen-activated protein kinase (MAPK)	Mehrabi et al. (2006a)	***PDS***	Phytoene desaturase-Carotenoid biosynthesis	Lee et al. (2015a,b)
***Hog1***	Mitogen-activated protein kinase (MAPK)	Mehrabi et al. (2006b)	***ChlH***	Magnesium chelatase sub-unit H-Chlorophyll biosynthesis	Lee et al. (2015a,b)
***STE11***	MAPK kinase kinase	Kramer et al. (2009)	***TaR1***	Homeodomain protein	Lee et al. (2015a,b)
***STE50***	Scaffold protein for MAPK signalling	Kramer et al. (2009)			
***STE12***	Transcription factor target of MAPK signalling	Kramer et al. (2009)			
***STE7***	MAPK kinase	Kramer et al. (2009)			
***Alg2***	Protein N-glycosylation	Motteram et al. (2011)			
***Gpa1***	G-protein alpha sub-unit	Mehrabi et al. (2009)			
***Gpa3***	G-protein alpha sub-unit	Mehrabi et al. (2009)			
***Gpb1***	G-protein beta sub-unit	Mehrabi et al. (2009)			
***Tpk2***	Protein kinase A catalytic sub-unit	Mehrabi and Kema (2006c)			
***Bcy1***	Protein kinase A regulatory sub-unit	Mehrabi and Kema (2006c)			
***3LysM***	Chitin binding effector protein	Marshall et al. (2011)			
***MCC1***	c-type cyclin	Choi and Goodwin (2011)			
***Wor1***	Transcription factor	Mirzadi Gohari et al. (2014)			
